# Load, pressure, rubble pile geometry and video data from model-scale tests on shallow water ice-structure interaction

**DOI:** 10.1016/j.dib.2022.108580

**Published:** 2022-09-13

**Authors:** Ida Lemström, Arttu Polojärvi, Otto Puolakka, Jukka Tuhkuri

**Affiliations:** Aalto University, School of Engineering, Department of Mechanical Engineering P.O. Box 14300, Aalto FI-00076, Finland

**Keywords:** Ice mechanics, Ice-structure interaction, Ice load, Model-scale tests, Offshore structures

## Abstract

This paper presents the data from model-scale experiments on shallow water ice-structure interaction. The data is related to the original research article ‘Model-scale tests on ice-structure interaction in shallow water: Global ice loads and the ice loading process’ Lemström et al.(2022)[1]. During the conducted experiments, a ten-meter wide initially intact ice sheet was pushed against a sloping structure of the same width. As the ice failed against the structure, a grounded rubble pile accumulated in front of it. The structure consisted of ten identical one-meter-wide segments and the horizontal load on each of these segments was measured independently with load cells. These measurements are presented as load-time datasets. The horizontal load acting on the false bottom was measured with load cells and are also presented as load-time datasets. Furthermore, the ice pressure on two of the segments was measured with tactile sensors. These pressure measurements are presented as array-based pressure-time datasets. Video footage filmed from two different video angles is published. In addition, the coordinates of the rubble pile geometries at the end of each experiment are published. The data includes the top and side rubble pile geometries. In total, seven experiments were conducted. The data can be used by researchers, engineers and designers who work with ice structure interaction related issues in order to, for instance, optimize the design of offshore structures, improve ice load predictions or develop future experiments and simulations.

## Specifications Table

**Table:** 

Subject	Ocean and Maritime Engineering
Specific subject area	Ice Mechanics
Type of data	Text file
	MP4 Video file
How data were acquired	The structure load data was measured with uni-axial HBM PW29C3 load cells and the bottom load data with Tedea-Huntleigh Model 620 load cells. The load outputs are load-time values. The pressure data was acquired with Tekscan #5400N tactile sensors and the outputs are array-based pressure-time data. The video footage was acquired with GoPro Hero 8 black action cameras. The rubble pile geometry data is determined visually.
Data format	Raw
	Video
Description of data collection	Model-scale experiments on the ice-structure interaction process in shallow water were performed by pushing a 10 m wide ice sheet against an inclined structure of the same width. The structure consisted of ten identical segments and the horizontal load on each segment was independently measured with a uni-axial load cell. In addition, the local pressure was measured with tactile sensors from two of the segments. The experiments were filmed with action cameras from two different directions.
Data source location	Institution: Aalto University
	City/Town/Region: Espoo
	Country: Finland
Data accessibility	Repository name: Zenodo
	Data identification number: https://doi.org/10.5281/zenodo.6524282
	Direct URL to data: https://zenodo.org/record/6524282#.YnVuLS8RqL1
Related research article	I. Lemström, A. Polojärvi, J. Tuhkuri, Model-scale tests on ice-structure interaction in shallow water: Global ice loads and the ice loading process, Marine Structures. 81 (2022). https://doi.org/10.1016/j.marstruc.2021.103106

## Value of the Data

• The presented load and pressure data provide new experimental insight on the shallow water ice-structure interaction process.
• The data can benefit researchers, engineers and designers who work with ice-structure interaction related issues.
• The data can be used to improve the ice load predictions on wide, inclined structures, to optimize the design of offshore structures, or to develop future experiments and numerical simulations.

## 1 Data Description

**Fig. 1:**
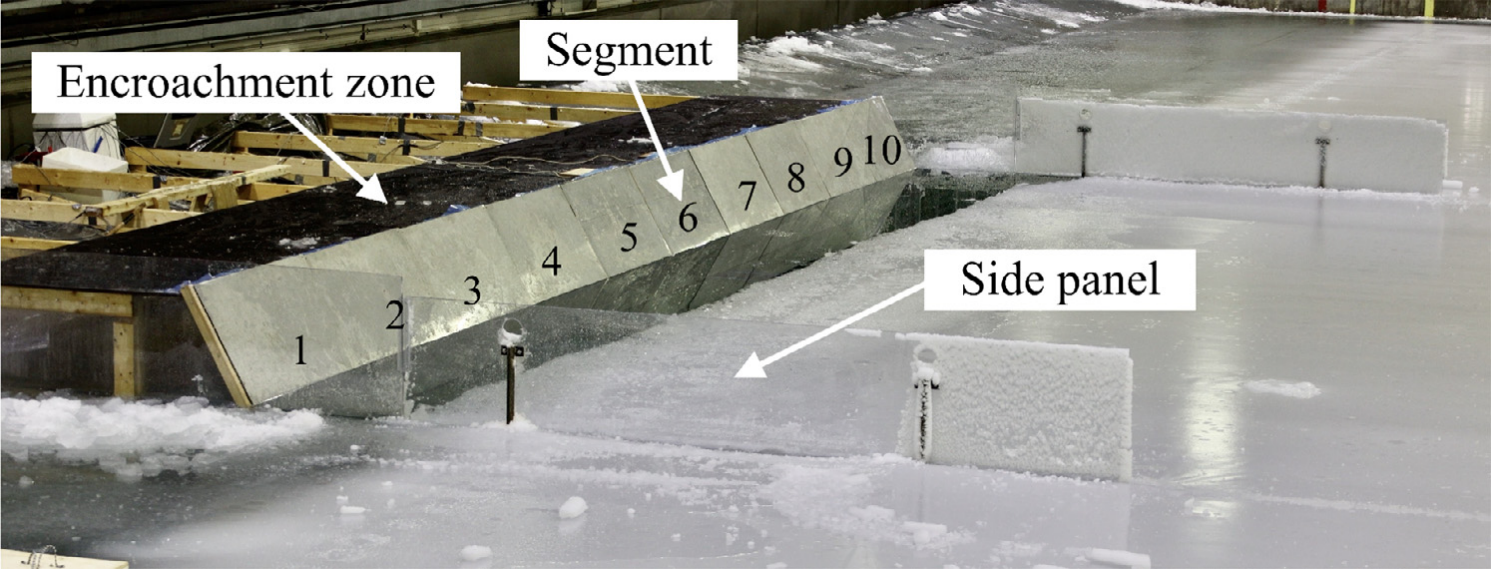
The general overview of the experimental set-up and segment numbering. The segments are numbered from 1 to 10 according to the figure. The figure is reproduced from Lemström et al. [1].

The data published is from seven model-scale ice-inclined structure interaction experiments performed in Aalto Ice Tank (Fig. 1). The case ID:s are 1–7 and the ice properties for each case are presented in Table 1.

**Table 1:** The ice properties and experimental parameters for each of the seven experiments. $H_{i}$ is the ice thickness, $\rho_{i}$ is the ice density, $\sigma_{f}$ is the flexural strength of the ice, $\sigma_{c}$ is the compressive strength of the ice, E is the elastic modulus and $L_{max}$ is the total length of ice pushed against the structure.

Case ID	$H_{i}$ [mm]	$\rho_{i}$ [kg/m^3^]	$\sigma_{f}$ [kPa]	$\sigma_{c}$ [kPa]	E [MPa]	$L_{\text{max}}$ [m]
1	50	926	44	46	107	23.2
2	52	928	50	51	112	19.7
3	52	934	42	54	103	21.6
4	50	916	109	121	387	25.3
5	51	920	108	130	328	25.9
6	52	915	211	214	2023	25.7
7	52	909	197	261	1529	24.6

**Fig. 2:**
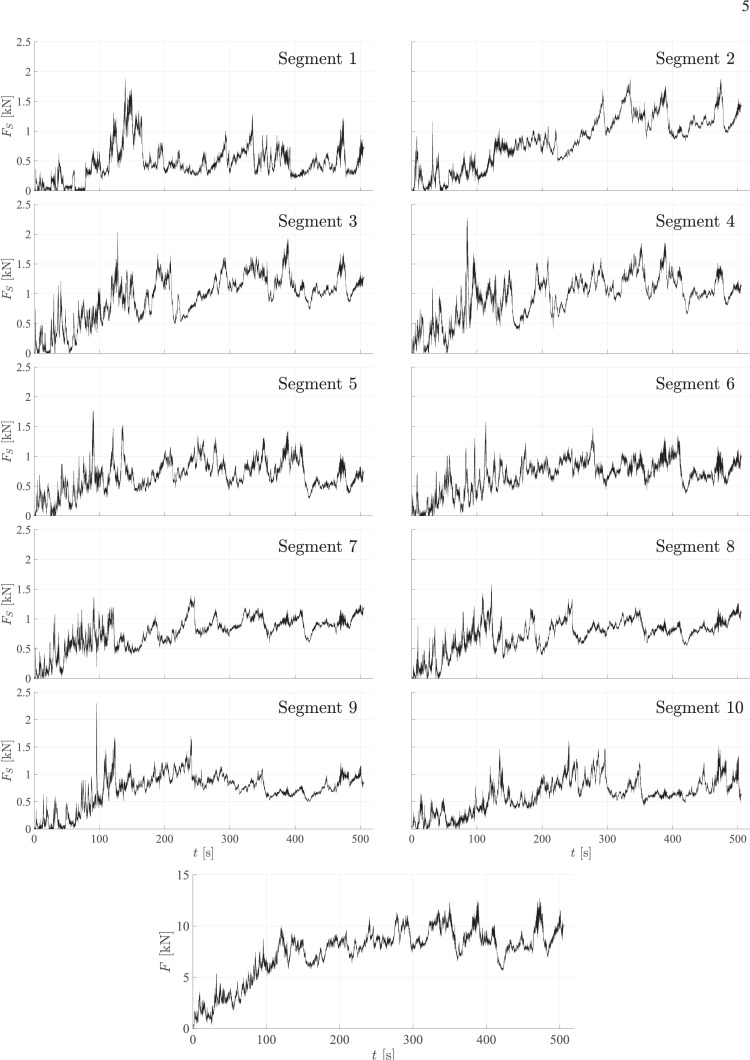
The horizontal segment ice load, $F_{S}$, measured independently from each of the ten segments for case ID 4, and the horizontal global load, F, as a function of the time, t. The global load is defined as the sum of all the segment load records.

For each case, load-time datasets and two video files from the experiment are published. The file with the filename ***caseA_structure_load.txt***, where A stands for the case ID, includes the load data measured from each of the ten individual segments of the structure. The first column in the file gives the time in seconds [s] and columns 2–11 include the horizontal ice load in Newtons [N] measured from segments 1–10, respectively. The columns are tab separated. The segments are numbered from one to ten according to Fig. 1. Fig. 2 illustrates the structure load data by showing the horizontal ice load, $F_{S}$, on each of the ten segments as a function of time, t, for case ID 4. In addition, horizontal global load, F, taken as the sum of the loads on the ten segments is presented in the undermost figure.

**Fig. 3:**
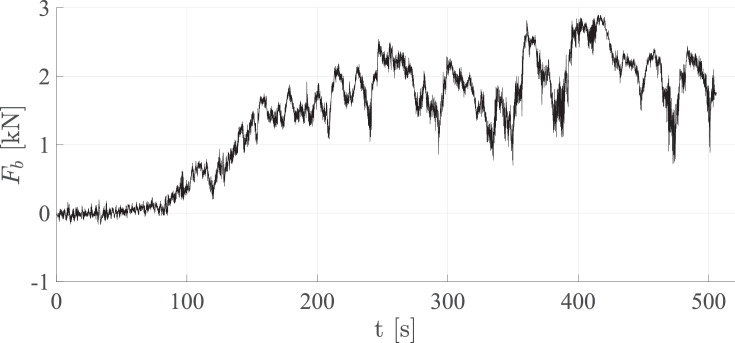
The horizontal ice load on the bottom, $F_{b}$, as a function of the time, t, for case ID 4.

Files with the filename ***caseA_bottom_load.txt*** include the load data measured from the bottom. The first column shows the time in seconds [s] and columns 2–5 show the horizontal load in Newtons [N], measured with four load cells attached to the false bottom. The columns are tab separated. Fig. 3 shows the horizontal ice load measured from the bottom, $F_{b}$, as a function of the time, t. $F_{b}$ is taken as the sum of the loads measured with the four load cells. The load data for both the structure and the bottom start at the instance of the experiment at which the ice sheet first hits the structure and ends when the experiment ends.

The files named ***caseA_pressure_segB.csv*** include the data measured from segments 5 and 6 by using tactile sensors. Only these two segments were instrumented with the tactile sensors. B stands for the segment number from which the data was measured. The pressure data is published in CSV format that the TekScan software gives as an output. The header of the data file presents information of the tactile sensor and the measurements. The measured pressure values are then given as comma separated 52 × 34 matrices, measured for each timestep recorded. The frame number and the timestamp of the measured pressure matrix is given before each matrix. In raw format, the pressures measured from the top row of sensels are given in the first column of the matrix and the pressures measured from the bottom row of sensels are given in column 34. Thus, the matrix should be transposed to obtain the describe the direction of the tactile sensor. The pressure data gives the pressure normal to the structure for each sensel as measured along the inclined structure. Due to technical challenges in the calibration of the tactile sensors, the raw pressure data should only be used in, for example, normalized form, and not interpreted as absolute pressure values. Due to technical problems, only the tactile sensor measurements for cases 2, 5 and segment 6 of case 4 were available and published. Table 2 shows the frames of the instances when the experiments starts and ends. These offsets can be used to synchronize the data with the load and video files.

**Table 2:** Offsets for the pressure data. The data indicate frames for the beginning and end of the experiments. The start frame corresponds to the instance when the ice first hits the structure and the end frame the instance when the experiment ends. These offsets can be used to synchronize the pressure data with the load and video files.

Case ID	Start frame	End frame
2	34,911	72,734
4	22,011	70,585
5	27,299	77,025

The video files are in MP4 format and named ***caseA_videoC.mp4***, where C stands for the camera view. The camera view C = 1 is filmed directly from above the structure, whereas the camera view C = 2 shows a general view of the experiment. The video file starts and ends at the same instance as the load files. During the experiments, GoPro cameras were used to obtain video footage.

**Fig. 4:**
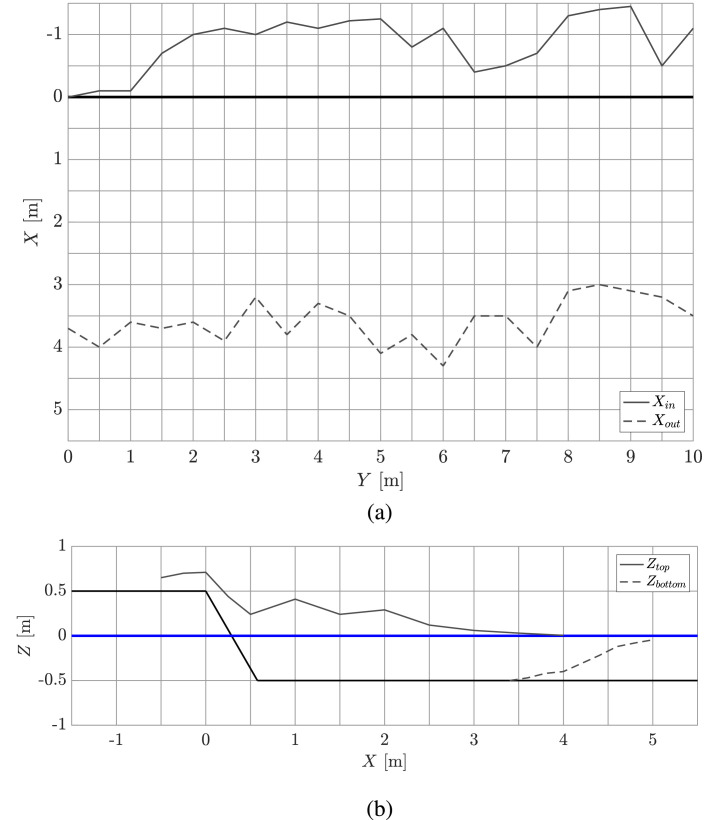
Illustration of the (a) top and (b) side rubble pile profile at the end of experiment with the case ID 5. The figure also shows the coordinate system and origin used in the rubble pile profile data files.

The rubble pile geometry data is also published. The files named ***caseA_geometry_above.txt, caseA_geometry_side_top.txt*** and ***caseA_geometry_side_bottom.txt*** include the coordinates for the top pile profiles, above-water side and below-water side rubble pile profiles, respectively, at the end of each experiment. Fig. 4 presents the top and side views of the rubble pile of case 5. The figure also shows the coordinate system and origin used in the data files. In the file ***caseA_geometry_above.txt***, the first column describes the Y-coordinate and columns 2-3 describe the X-coordinates for the inner and outer edges of the rubble pile, $X_{i}n$ and $X_{o}ut$, respectively (Fig. 4(a)). The coordinates of the top rubble pile profiles are determined visually from the video footage. In the files ***caseA_geometry_side_top.txt*** and ***caseA_geometry_side_bottom.txt***, the first column describes the X-coordinate and the second column describes the Z-coordinates of the above- and below-water side pile profile, $Z_{t}op$ and $X_{b}ottom$, respectively (Fig. 4(b)). The above-water side profiles describe the average of three profiles as measured after each experiment: one from the both ends and one from the middle of the structure (points Y = 0 m, Y = 5 m and Y = 10 m of Fig. 4(a)). The side profiles below water illustrate the profiles at point Y = 10 m only. The below-water side profiles are determined visually from the video footage. The below-water side profile for case 2 is missing due to lacking video footage.

## 2 Experimental Design, Materials and Methods

### 2.1 Experimental set-up

**Fig. 5:**
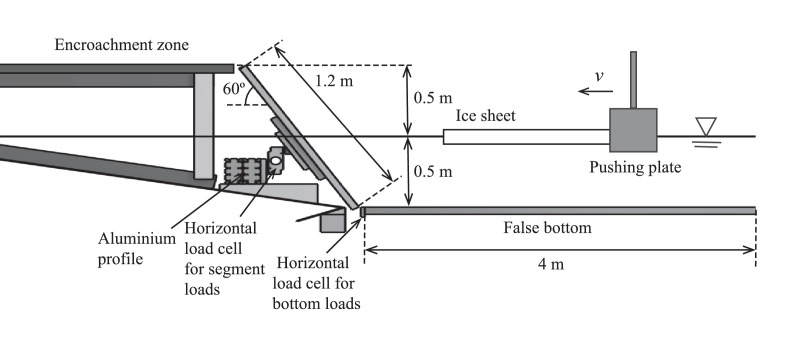
The experimental set-up and main dimensions. The figure is reproduced from Lemström et al. [1].

The model-scale experiments were conducted in the Aalto Ice Tank. The experimental set-up and the instrumentation used are presented in Figs. 1 and 5, and Table 3, respectively. The structure was 10 m wide, 1 m tall and had an inclination angle of 60${}^{\circ }$. The basin was equipped with a retrievable false bottom so that the water depth of the structure and its freeboard were 0.5 m. In the experiments, an intact ice sheet was pushed against the structure with constant velocity of 0.05 m/s. The ice sheet strip pushed against the structure had the same width as the structure. The structure was confined in between two vertical plexi-glass panels, restricting sideways motion of the ice. The structure consisted of ten identical one-meter-wide segments. The segments were mounted on an aluminium beam which was installed to the ice basin (Fig. 5). The segments consisted of plywood plates with the thickness 40 mm and had additional 30 mm thick plywood and aluminium plates behind them to make them stiff. In addition, the segments were coated with 1 mm thick sheets of stainless steel. The friction coefficient between the ice and the structure surface was measured to be approximately 0.1. The horizontal load on each of the segments was independently measured with S-shaped uni-axial load cells, installed between the segments and the beam.

**Table 3:** Summary of the instrumentation utilized to acquire the data.

Type of instrument	Model	Location	Range	Resolution [Hz]	Function
Data acquisition system	QuantumX MX410B	–	–	–	data collection
Load cell	HBM: PW29C3	segments	0–1000 kg	2400	loads on segments
Load cell	Tedea-Huntleigh: Model 620	false bottoms	0–500 kg	2400	loads on bottoms
Tactile sensor	Tekscan sensor #5400N	segments 5 and 6	28–648 kPa	96	local pressures
Camera	GoPro Hero 8 black	Ice Tank ceiling	–	–	video image

The retrievable bottom extended 4 m away from the structure (Fig. 5). The bottom was fabricated of plywood and it was covered with polypropylene carpet. The friction coefficient between the ice and the bottom was measured to be approximately 0.55. The horizontal load induced on the bottom was measured by S-shaped, uni-axial load cells, which were pre-stressed to the side of the basin with springs. The loads in this case were measured with four load cells. Furthermore, the structure had a 1.25 m long horizontal encroachment zone installed on top of it (Fig. 1).

The local ice pressures from segments 5 and 6 (Fig. 1) were measured with tactile sensors. The tactile sensors had the dimensions of 0.88 m × 0.58 m (width × height) and they consisted of 52 rows and 34 columns of sensels. Each sensel had an area of 17 mm × 17 mm. In order to be protected from water and ice abrasion, the tactile sensors were installed behind the steel coating of the segments. The tactile sensors were located in the middle of the segments, reaching 0.29 m below and above the waterline along the sloping structure.

### 2.2 Ice properties and test procedure

The model ice used in the experiments was granular and produced with the standard ice production techniques of the Aalto Ice Tank [2]. The ice was grown by spraying, during which a fine mist of 0.3% ethanol-doped water was continuously and uniformly sprayed above the basin to form ice, layer-by-layer, until the desired ice thickness was reached. The ambient air temperature during the spraying was −10⋯−16 ${}^{\circ }$C. After the spraying, the ice was tempered to achieve the target ice properties.

Table 1 presents the ice thickness, $H_{i}$, ice density, $\rho_{i}$, flexural strength, $\sigma_{f}$, compressive strength, $\sigma_{c}$, elastic modulus, E, and the maximum length of ice pushed against the structure, $L_{\text{max}}$, for each of the seven experiments performed. The target ice thickness for all experiments was 50 mm. The flexural and compressive strength in tests 1–3 was ∼50 kPa, whereas for tests 4–5 and 6–7, the strength of the ice was approximately two and four times higher, respectively. The ice-ice friction coefficient was measured separately in all experiments and varied between 0.1 and 0.15.

The flexural strength of the ice was measured in-situ with the cantilever beam method, as recommended by the International Towing Tank Conference (ITTC) [3]. The compressive strength of the ice was also measured in-situ, by axially loading short cantilever beams until their failure while recording the force. The compressive strength was then taken as $\sigma_{c}=f/bH_{i}$, where f is the force at failure, b the width of the ice beam, and $H_{i}$ the ice thickness. The beam width and length were equal to the ice thickness when testing the compressive strength. The infinite plate test, as recommended by the ITTC, was used to measure the elastic modulus [3].

## Ethics Statement

This article does not involve the use of human or animal subjects.

## CRediT authorship contribution statement

**Ida Lemström:** Conceptualization, Methodology, Validation, Formal analysis, Investigation, Data curation, Writing – original draft, Visualization. **Arttu Polojärvi:** Conceptualization, Methodology, Supervision, Writing – review & editing. **Otto Puolakka:** Methodology, Writing – review & editing. **Jukka Tuhkuri:** Conceptualization, Writing – review & editing.

## Declaration of Competing Interest

The authors declare that they have no known competing financial interests or personal relationships which have, or could be perceived to have, influenced the work reported in this article.

## Data Availability

Load, pressure, rubble pile geometry and video data from model-scale tests on shallow water ice-structure interaction (Original data) (Zenodo).
